# Hypersensitive Response of Plasmid-Encoded AHL Synthase Gene to Lifestyle and Nutrient by *Ensifer adhaerens* X097

**DOI:** 10.3389/fmicb.2017.01160

**Published:** 2017-06-28

**Authors:** Yanhua Zeng, Yanli Wang, Zhiliang Yu, Yili Huang

**Affiliations:** ^1^Zhejiang Provincial Key Laboratory of Organic Pollution Process and Control, Department of Environmental Science, College of Environmental and Resource Sciences, Zhejiang UniversityHangzhou, China; ^2^College of Biotechnology and Bioengineering, Zhejiang University of TechnologyHangzhou, China

**Keywords:** *Ensifer adhaerens*, hypersensitive, plasmid-encoded, AHL synthase, lifestyle, nutrient

## Abstract

It is known that some bacteria, especially members of the family *Rhizobiaceae*, have multiple *N*-acyl homoserine lactones (AHL) synthase genes and produce multiple AHL signals. However, how bacteria selectively utilize these multiple genes and signals to cope with changing environments is poorly understood. *Ensifer adhaerens* is an important microorganism in terms of biotechnology, ecology and evolutionary. In this study, we investigated the AHL-based QS system of *E. adhaerens* X097 and its response to different lifestyles or nutrients. Draft genome sequence data indicated that X097 harbored three distinct AHL synthase genes (*ensI1, 2, 3*) and seven *luxR* homologs, which was different from other *E. adhaerens* strains. *In vitro* expression indicated that plasmid-encoded *ensI1* and *ensI2* directed production of multiple AHLs, while chromosome-encoded *ensI3* only directed production of C14-HSL. Predicted three dimensional structure of EnsI3 was quite different from that of EnsI1 and EnsI2. X097 produced different AHL profiles in Luria-Bertani (LB) and NFB medium, under biofilm and planktonic lifestyle, respectively. Notably, expression of *ensI1* and *ensI2* but not *ensI3* is hypersensitive to different lifestyles and nutrients. The hypersensitive response of plasmid-encoded AHL synthase genes to different culture conditions may shed a light on the phylogenetic development of AHL synthase genes in *Rhizobiaceae* family.

## Introduction

Quorum sensing (QS) is a cell density-dependent communication system in bacteria (Asfahl and Schuster, 2017). *N*-acyl homoserine lactones (AHLs)-based QS system is quite common among bacteria belonging to the *Rhizobiaceae* family, wherein many strains represent some of the best studied QS model organisms, such as *Agrobacterium tumefaciens, Rhizobium leguminosarum, Rhizobium etli*, and *Sinorhizobium meliloti* (Wisniewski-Dyé and Downie, 2002; Marketon et al., 2003; White and Winans, 2007). Many of the QS-regulated physiological traits including plasmid transfer, symbiotic nitrogen fixation and surface polysaccharide production (Daniels et al., 2002; Danino et al., 2003; Marketon et al., 2003; White and Winans, 2007) which play important roles in the rhizosphere. In a recent report, phylogenomic analysis showed that two major superclades were observed within the family *Rhizobiaceae*, which corresponded to the *Rhizobium*/*Agrobacterium* and *Shinella*/*Ensifer* groups. The type species of the *Ensifer* genus, *Ensifer adhaerens*, was an outlier in the latter group which separated from the rest of the *Ensifer* strains (Ormeño-Orrillo et al., 2015). This emphasizes the distinctive taxonomical status of *E. adhaerens*. To our knowledge, AHL-producing *E*. *adhaerens* has never been mentioned in previous studies until we found its prevalence in freshwater biofilm, soil and plant rhizosphere in our recent works (Huang et al., 2012, 2013a; Zeng et al., 2014).

The genus *Ensifer* was first proposed by Casida (1982), with the single species *E. adhaerens* sp. nov., and strain A (=ATCC 33212). However, more research on *E. adhaerens* has only emerged in recent 10 years, which mainly focused on plant–bacterial interaction and bioremediation. As a non-*Agrobacterium* species, *E. adhaerens* strain OV14 could transfer genes into several plant genomes via horizontal gene transfer when equipped with Ti plasmid based virulence machinery (Rudder et al., 2014). Besides, *E. adhaerens* has also been reported to have plant growth promoting and nitrogen-fixing abilities (Zhou et al., 2013). There are field and experimental evidences of the potential of *E. adhaerens* for bioremediation of recalcitrant xenobiotic compounds (Xu et al., 2015). Considering its significant ecological role and that AHL-driven QS circuit has never been systematically studied, the exploration of QS system in *E. adhaerens* would provide basic information for research on QS-controlled physiological activity.

In soil, *E. adhaerens* exists either as free-living bacteria or as nitrogen-fixing symbionts inside root nodules, which implies two basic environmental factors, lifestyle and nutrient. Compared with the planktonic counterpart, bacteria in biofilm respond as a community which promotes cell–cell interactions (Remis et al., 2010). There is no doubt that rhizobia frequently face fluctuating environment and that these bacteria must possess essential regulatory mechanisms like QS system to ensure survival and successful symbiosis. There is increasing evidence that the expression of QS genes and AHL production can be affected by various environmental factors (Sanchez-Contreras et al., 2007; Schwartz et al., 2007; Pérez-Montaño et al., 2011; Lee and Zhang, 2015). However, previous studies were mainly focused on the effect of environmental factors on each AHL synthase gene separately. From the growing genome data and increasing report cases, it is notable that there are multiple AHL synthase genes coexist in a strain in the *Rhizobiaceae* family quite frequently (Case et al., 2008). It is intriguing that how bacteria selectively utilize multiple AHL synthase genes and signals to cope with changing environment.

In our previous study, a novel AHL-based QS system in *Rhizobiaceae* family was identified in the black soil isolate *E. adhaerens* X097 (Huang et al., 2013a). Strain X097 harbored two AHL synthase genes and produced multiple AHL molecules including 3-oxo-C6-HSL and 3-oxo-C8-HSL, and some others remained to be identified. In this study, we analyzed the complete QS elements of strain X097. The expression of AHL synthase genes and AHL profiles of X097 under planktonic and biofilm lifestyle, in LB or NFB medium were compared. This is the first comprehensive study of the QS system in *E. adhaerens* species and its response to different lifestyles and nutrients.

## Materials and Methods

### Bacterial Strains, Media and Growth Conditions

Bacterial strains and plasmids used in this study are listed in **Table 1**. *E. adhaerens* X097 was routinely cultivated in Luria-Bertani (LB) medium at 28°C. The nitrogen-free base medium (NFB) which represents nutrient deficient supply was prepared according to the literature (Zhou et al., 2013). AHL biosensor strain *A. tumefaciens* A136 (pCF218) (pCF372) was grown in LB medium supplemented with appropriate antibiotics (spectinomycin, 50 μg⋅mL^-1^; tetracycline, 4.5 μg⋅mL^-1^) at 28°C. All *Escherichia coli* strains were grown in LB medium at 37°C. When required, the growth medium was supplemented with ampicillin (100 μg⋅mL^-1^) or kanamycin (50 μg⋅mL^-1^).

**Table 1:** Strains and plasmids used in this study.

Strain or plasmid	Relevant characteristics	Source
**Strains**		
*Ensifer adhaerens* X097	wild type	Huang et al., 2013a
*Agrobacterium tumefaciens* A136(pCF218)(pCF372)	traI-lacZ fusion; AHL biosensor	McLean et al., 1997
*Escherichia coli* DH5α	F*^-^ hsdR17 endA1 thi-1 gyrA96 relA1 supE44*Δ*lacU169 (*φ80*dlacZ*ΔM15*)*	TransGen Biotech
*Escherichia coli* BL21(DE3)	F^-^ *ompT hsdS_B_ (r_B_*^-^ *m_B_*^-^*) gal dcm* (DE3)	TransGen Biotech
**Plasmids**		
pET-28b(+)	T7 expression vector	Novagen
pET28b(+)-*ensI1*	pET-28b (+) containing AHL synthase gene *ensI1*	This study
pET28b(+)-*ensI2*	pET-28b (+) containing AHL synthase gene *ensI2*	This study
pET28b(+)-*ensI3*	pET-28b (+) containing AHL synthase gene *ensI3*	This study

### Draft Genome Sequencing, Annotation and Identification of QS Elements in *E. adhaerens* Genomes

To get a full picture of AHL-based QS system in *E. adhaerens*, the draft genome of X097 was sequenced. Genomic DNA was extracted by using the Bacterium Total DNA Isolation Kit (Simgen, China). Total DNA was subjected to quality control using 1% agarose gel electrophoresis and quantified by Qubit3.0 Fluorometer (Invitrogen, United States). The genome of X097 was sequenced with MPS (massively parallel sequencing) Illumina technology. Library construction and sequencing were performed at the Beijing Novogene Bioinformatics Technology Co., Ltd. The filtered reads were assembled by SOAPdenovo^1^ to generate scaffolds (Li et al., 2008). Genome annotation was performed by whole genome blast search (*E*-value less than 1e-5, minimal alignment length percentage larger than 40%) against six databases (Altschul et al., 1990) including KEGG (Kyoto Encyclopedia of Genes and Genomes), COG (Clusters of Orthologous Groups), NR (Non-Redundant Protein Database databases), Swiss-Prot, GO (Gene Ontology) and TrEMBL.

Genome sequences in two other strains of *E. adhaerens*, OV14 and Casida A (abbreviated as CA), which were available on NCBI GenBank database under accession numbers CP007236-CP007239 and CP015880-CP015882, were also analyzed for identification of QS elements. Basic QS elements including *luxI* and *luxR* homologs in the genomes of three *E. adhaerens* strains were identified using either keywords (acyl homoserine lactones synthase or autoinducer for *luxI*, and LuxR family transcriptional regulator for *luxR*) searching or BLASTX program.

### Survey and Phylogenetic Analysis of AHL Synthase Genes among *Rhizobiaceae* Family

All AHL synthase genes from *Rhizobiaceae* family with complete genome sequences in NCBI Genome Database were obtained by using the keywords (acyl homoserine lactones synthase or autoinducer) searching method. Genome data were collected by date January 31st, 2015. MEGA 5.1 program was used to construct phylogenetic tree from these AHL synthases (Tamura et al., 2011). Sequences were aligned using the algorithm ClustalW implemented in MEGA 5.1 (Thompson et al., 1994), and then subjected to phylogenetic analysis using neighbor-joining method (Saitou and Nei, 1987).

### Cloning and *In Vitro* Expression of AHL Synthase

Three AHL synthase genes of strain X097 were cloned and expressed in *E. coli* BL21 (DE3) using the expression vector pET28b (+) according to manufacturer’s instruction (Novagen, United States). The primers used were listed in Supplementary Table S1. The expression of three AHL synthase genes was induced by the addition of 0.5 mM isopropyl-β-D-thiogalactopyranoside (IPTG) at 28°C when the OD_600_ of recombinant cultures reached 0.4–0.6. Over-expressed AHL synthases were visualized by 12% SDS-PAGE as described by Sambrook and Russell (2001).

### Prediction of Three Dimensional Structures of AHL Synthases

Three dimensional (3D) structure of AHL synthase of X097 was predicted by using SWISS-MODEL, a fully automated protein structure homology-modeling server^2^ according to the standard protocol (Arnold et al., 2006). The PyMOL Molecular Graphics System (version 1.2r3pre) was used to view, render and animate 3D molecular structures of AHL synthase (DeLano, 2002). The 3D structures of EnsI1 and EnsI2 were based on the experimental crystal structure of LasI of *Pseudomonas aeruginosa* (SMTL id 1ro5.1) (Gould et al., 2004), whereas the 3D model of EnsI3 was based on the crystal structure of TofI of *Burkholderia glumae* (SMTL id 3p2h.1) (Chung et al., 2011).

### TLC and GC-MS Analysis of AHL Profiles

*N*-acyl homoserine lactones were extracted from LB broth or agar plates using ethyl acetate (EA), and AHL profiles were analyzed by both TLC and GC-MS methods as previously described (Huang et al., 2013b).

### Growth Curve of X097 in LB and NFB Broth

The growth curve of X097 cultured in LB broth was drawn from monitoring the OD_600_ value every hour or two, using the Spectrophotometer (Metash, China). Due to the semi-transparent trait of X097 grown in NFB medium, the OD_600_ value was quite low (the maximum OD_600_ value is less than 0.11 even at stationary phase) and it introduced large instrument measurement error. So the growth curve of X097 in NFB broth was drawn from measuring the CFU (per milliliter) value. Fresh colony of X097 was first inoculated into NFB broth and incubated at 28°C for 5 days. 1 mL of this seed culture was re-inoculated into 100 mL of fresh NFB broth and incubated at 28°C, at a shaking speed of 200 rpm. The CFU number was calculated using plating method. All data above were taken from three replicates.

### Bacterial Cells Prepared from Different Lifestyles and Nutrients

Luria-Bertani and NFB medium represent rich and deficient nutrients supply. X097 grown in broth culture with shaking was referred to planktonic lifestyle, while X097 streaked on agar plates was referred to biofilm lifestyle. According to the growth curve in LB, planktonic cells were harvested by centrifugation at 16, 25, 40, and 64 h, representing the lag, log, late log and stationary phases for stage of growth, respectively. Biofilm cells were scraped from the agar plates at 16, 25, 40, and 64 h as well. For NFB medium, planktonic and biofilm cells were harvested at 8 and 12 days, respectively. To see the effect of nutrient on the expression of QS genes, both LB and NFB biofilm cells were harvested at 1, 2, 3, and 4 days, respectively. Due to the large difference in growth rate, the effect of nutrient in planktonic lifestyle was not analyzed here.

### Quantitative Real Time PCR

Quantitative real time PCR (qRT-PCR) was performed to compare the relative transcription level of three AHL synthase genes and *ensR3* gene under different lifestyles and nutrients. Total RNA was extracted using the RNAiso Plus Kit (TaKaRa, Dalian, China) according to the manufacturer’s protocol. The purity and quantity of all RNA samples were checked with a NanoDrop ND1000 spectrophotometer (Thermo Scientific, United States). Recombinant DNase I (RNase-free) (TaKaRa, Dalian, China) treatment was performed to remove the genomic DNA contamination in the total RNA. Reverse transcription was performed with 500 ng of total RNA at a final volume of 10 μL reaction using the PrimeScript^TM^ RT reagent Kit (Perfect Real Time) (TaKaRa, Dalian, China). The mixture was incubated for 15 min at 37°C for reverse transcription and then for 5 s at 85°C to inactivate the polymerase. The resultant cDNA samples were used as the templates for amplification of three AHL synthase genes and *ensR3* gene by using the SYBR *Premix Ex Taq*^TM^ Kit (Tli RNaseH Plus) (TaKaRa, Dalian, China) in Applied Biosystems StepOnePlus^TM^ Real-Time PCR System (Thermo Scientific, United States). 16S rRNA gene was served as the housekeeping gene to normalize the difference of cDNA amounts between different samples. Primer pairs used for qRT-PCR analysis are listed in Supplementary Table S1. Each reaction tube containing 5 μL of cDNA with 100-fold of dilution, 250 nM of each of the forward and reverse primers, 25 μL of 2× SYBR Green PCR Mix, 1 μL of ROX Reference Dye and nuclease-free water to a final volume of 50 μL. All reactions were performed in triplicates. The thermal cycler settings were programmed for 1 min at 95°C followed by 40 cycles consisting of 5 s at 95°C and 32 s at 60°C. At the end of the real-time PCR run, a dissociation stage was added to confirm specific product amplification. Negative controls with nuclease-free water as templates for each primer set were included in each run.

The data obtained were analyzed by using StepOne Software v2.3 (Life Technologies) which automatically set the cycle threshold (*C_T_*). The relative expression level of each target gene under different lifestyles or nutrients was calculated by the comparative *C_T_* method (Schmittgen and Livak, 2008). Mean difference of relative expression level of target gene between different culture conditions was statistically analyzed by ANOVA in Microsoft Office 2013. Primer efficiency was determined by the following method: Several dilutions of cDNA sample were assayed for each target gene in order to obtain a standard curve between the *C_T_* values and the log of the cDNA. The amplification efficiency of each primer pair was retrieved from the slope of the standard curve according to the formula *E* = 10^(-1/slope)^.

### Nucleotide Sequence Accession Number

Draft genome sequence of strain X097 has been deposited at DDBJ/ENA/GenBank under the accession number MRVD00000000. The version described in this paper is version MRVD01000000.

## Results

### Prevalence and Phylogeny of Multiple AHL Synthase Genes in Strains from Rhizobiaceae Family

*N*-acyl homoserine lactones synthase genes from isolates in the *Rhizobiaceae* family with whole genome annotations were retrieved from NCBI genome database and summarized in **Table 2**. The abundance and location of AHL synthase genes of individual bacterium among different species, even among different strains in the same species, were quite different. Of the 36 strains, 9 had AHL synthase genes only on plasmid, 6 only on chromosome, and 21 on both. On the other hand, among the 36 strains, 12, 13, 7, and 4 of them had one, two, three, and four AHL synthase genes, respectively. In total, about 67% strains contain 2 or more AHL synthase genes.

**Table 2:** Location and number of AHL synthases in different strains in *Rhizobiaceae* family searched by genome mining.

Bacterial species	Location and number of AHL synthase in different strains
*Agrobacterium fabrum*	P1
*Agrobacterium radiobacter*	C1P1
*Agrobacterium tumefaciens*	P1, P1, P2
*Agrobacterium vitis*	C1P3
*Ensifer adhaerens*	C1P1, C1P1, C1P2
*Rhizobium freirei*	P1
*Rhizobium gallicum*	C1P2
*Rhizobium* sp.	P4
*Rhizobium tropici*	P2
*Rhizobium etli*	C1P2, C1P1, C1P2, C1P3, C1P1
*Rhizobium leguminosarum*	C1P3,C1P2,C1,C1P1,C1P1
*Sinorhizobium fredii*	C1P1,C1,C1P1
*Sinorhizobium medicae*	C1P2
*Sinorhizobium meliloti*	C1,C1,C1P1,P1,C1P2,C1,C1,C1P1
*Neorhizobium galegae*	P1

*P, plasmid; C, chromosome. Different strains are separated by a comma.*

**FIGURE 1:**
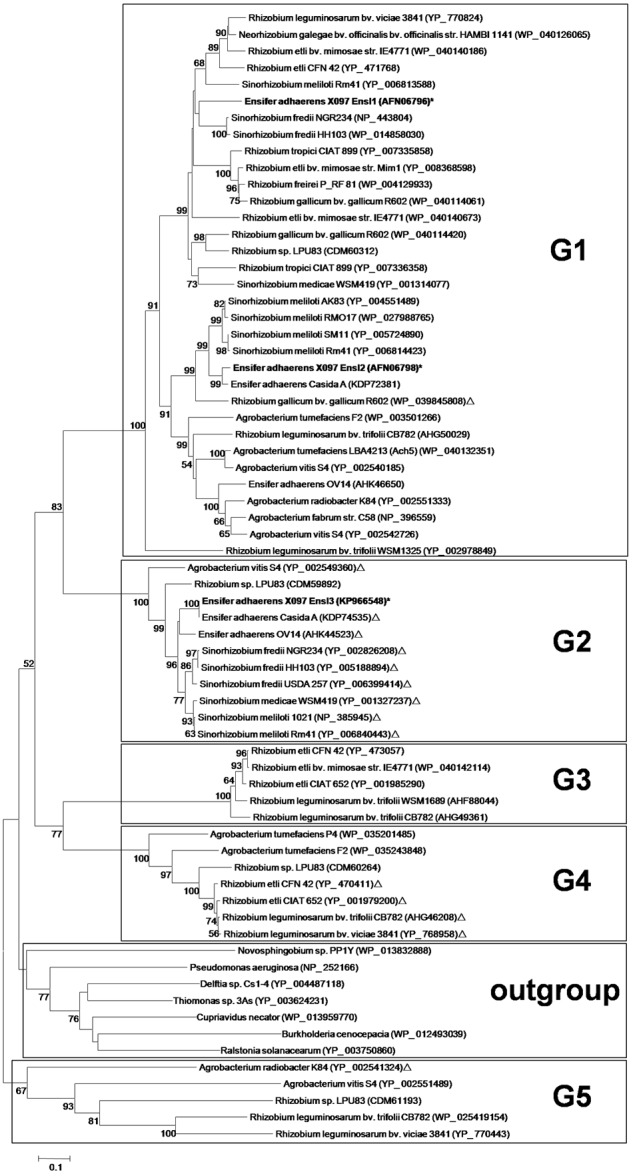
Phylogenetic neighbor-joining tree of all AHL synthases from isolates in the family *Rhizobiaceae* with whole genome annotations. The exception was synthases of strain X097, which were obtained from draft genome sequences. The tree was constructed by the amino acid sequence of each AHL synthase. AHL synthases on chromosome were marked by ‘Δ’ and AHL synthases on plasmid were printed with no markers. Genes from X097 were marked by ‘^∗^’. Bootstrap values are based on 1000 replicates and only values >50 are shown. Bar, 0.1 substitutions per nucleotide position. Black boxes mark the 5 groups defined in this study (G1–G5) and the outgroup (non-*Rhizobiaceae*).

A phylogenetic tree of these AHL synthase genes was constructed (**Figure 1**). According to the topology, we divided the phylogenetic tree into 5 groups (G1–G5). Interestingly, almost all AHL synthase genes clustered in group G1, G3, and G5 were plasmid-encoded, while AHL synthase genes clustered in G2 were mostly chromosome-encoded. There was only one exception gene that with different location in group G1, G2, and G5, respectively. Group G4 contained AHL synthase genes from both plasmid and chromosome. Interestingly, AHL synthase genes in G5 were more distant from the other groups than those AHL synthase genes from non-*Rhizobiaceae* family. The three AHL synthase genes of X097 did distribute in two distinct groups, with *ensI1* and *ensI2* in group G1 and *ensI3* in group G2.

### The Compositions of QS Elements in *E. adhaerens* Genomes Are Diverse

**FIGURE 2:**
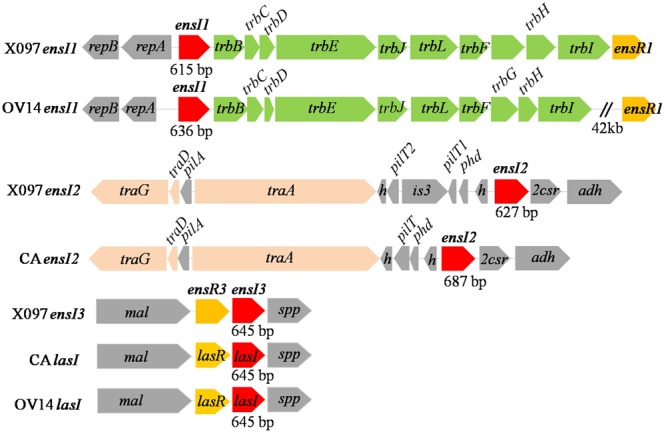
Gene arrangement of *luxI* homologs in the draft genome sequenced *E. adhaerens* X097, and whole genome sequenced *E. adhaerens* OV14 and CA. *traA-D-G*: gene coding for conjugal transfer protein TraA-D-G; *trbB-C-D-E-J-L-F-G-H-I*: gene coding for conjugal transfer protein TrbB-C-D-E-J-L-F-G-H-I; *repB/A*: gene coding for replicator protein RepB/A; *h*: gene coding for hypothetical protein; *pilA*: gene coding for pilus assembly protein; *pilT*: gene coding for twitching motility protein PilT; *is3*: gene coding for transposase IS3; *phd*: gene coding for prevent-host-death protein; *adh*: amidohydrolase gene; *2csr*: transcriptional regulatory gene in two component system; *mal*: malic enzyme gene; *spp*: gene coding for signal peptide protein.

16S rRNA gene of X097 shared 99% identity to that of OV14 and CA. There were 11 bp difference in 16S rRNA gene between X097 and OV14, and 3 bp between X097 and CA. By genome sequencing, we obtained a third AHL synthase gene *ensI3* and transcriptional regulator *ensR3* gene on the X097 genome, in addition to two known AHL synthase genes reported in our previous study (Huang et al., 2013a). In this study, the two genes were officially named *ensI1* and *ensI2*. Gene *ensI1* and *ensI2* were assumed located on plasmid according to the nearby plasmid specific genes, such as *trbB-C-D-E-J-L-F-G-H-I* and *traA-D-G* (**Figure 2**). The *ensI3* and *ensR3* genes were assumed on chromosome since we found that both strains OV14 and CA have *lasI* and *lasR* gene pair on chromosome, with high similarity to that of X097 (**Figure 2**). Upstream and downstream genes of *ensI3*/*ensR3* and *lasI*/*lasR* were also quite conserved in three strains (**Figure 2**). In total, there were three AHL synthase genes in X097. However, we only found two AHL synthase genes in the genome of both OV14 and CA (**Figure 2**). There were 7, 6, and 5 *luxR* homolog genes in the genome of X097, OV14 and CA, respectively. This indicated the variety of QS systems among different strains of *E. adhaerens*. So far, the basic genomic elements for AHL-based QS system in X097 contain 3 AHL synthase genes and 7 *luxR* homolog genes.

The EnsI3 of X097 was 89.3 and 100% similar to its homolog in OV14 and CA, respectively, while the EnsR3 of X097 was 88.2 and 100% similar to its homolog in OV14 and CA, respectively. Within X097, EnsI3 was 25.7% similar to EnsI1 and 27.4% similar to EnsI2. An identity matrix of these proteins was included in Supplementary Table S2.

### Three AHL Synthases Produce Different AHL Profiles *In Vitro*

**FIGURE 3:**
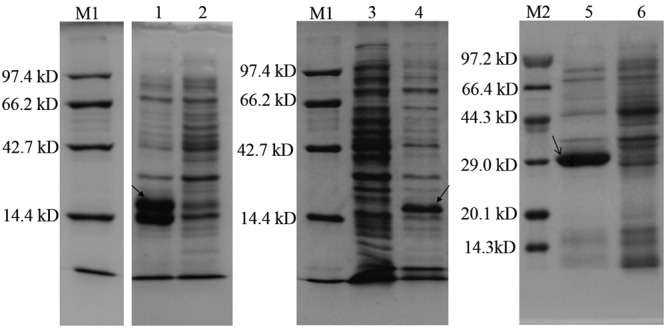
SDS-PAGE assay of three target AHL synthases expressed in *Escherichia coli*. 1, induced expression of EnsI1; 2, non-induced expression of EnsI1; 3, non-induced expression of EnsI2; 4, induced expression of EnsI2; 5, induced expression of EnsI3; 6, non- induced expression of EnsI3. M1and M2 are standard protein markers.

Three AHL synthase genes were successfully cloned into *E. coli* BL21 (DE3) and expression of the recombinant proteins were verified by SDS-PAGE. Significant bands at 22.75, 22.85, and 23.81 KD were found in IPTG induced BL21 (DE3)/pET28b (+)-*ensI1*, -*ensI2* and -*ensI3* cells, respectively (**Figure 3**). As expected, strain BL21 (DE3) containing recombinant plasmids pET28b (+)-*ensI1*/*ensI2*/*ensI3* produced AHL as detected by the reporter strain *A. tumefaciens* A136. As shown in Supplementary Figure S1A, the expression products of three AHL synthases in the heterologous *E. coli* were all positive in A136 bioassay, which was reflected by the blue colors on the agar plates pre-spreaded with X-gal solution. TLC analysis showed that EnsI1 directed the production of 3-oxo-C8-HSL and C8-HSL, while EnsI2 was responsible for the production of multiple AHL signals including 3-OH-C6-HSL, 3-oxo-C8-HSL, C8-HSL and some unknown AHLs (Supplementary Figure S1B). AHLs synthesized by EnsI3 were not detectable on TLC. All extracts were subjected to GC-MS analysis. Results revealed the production of C14-HSL by EnsI3, 3-oxo-C8-HSL and C8-HSL by EnsI1 that detected on TLC, C8-HSL by EnsI2 that detected on TLC and C10-HSL by EnsI2 that not detected on TLC (**Table 3** and Supplementary Figure S2). The AHL profiles produced by three AHL synthases were summarized in **Table 3**.

**Table 3:** *N*-acyl homoserine lactones (AHL) profiles expressed by three AHL synthases, and in different medium or lifestyle analyzed by TLC and GC-MS methods.

		TLC	GC-MS
*In vitro* (expressed in the heterologous *E. coli*)	EnsI1	3-oxo-C8-HSL; C8-HSL	3-oxo-C8-HSL; C8-HSL
	EnsI2	3-OH-C6-HSL; 3-oxo-C8-HSL; C8-HSL; and some ND AHL	C8-HSL; C10-HSL
	EnsI3	ND	C14-HSL
*In vivo* (expressed in strain X097)	LB broth	3-oxo-C8-HSL; and 2 ND AHL	3-oxo-C8-HSL; C8-HSL
	LB biofilm	3-oxo-C8-HSL; and 2 ND AHL	3-oxo-C8-HSL; C8-HSL 3-oxo-C6-HSL; C6-HSL
	NFB broth	C6-HSL	C8-HSL
	NFB biofilm	C6-HSL	C6-HSL; 3-oxo-C6-HSL

*ND means not determined signals.*

### Three Dimensional Structure of AHL Synthase Predicted by Swiss-Model

**FIGURE 4:**
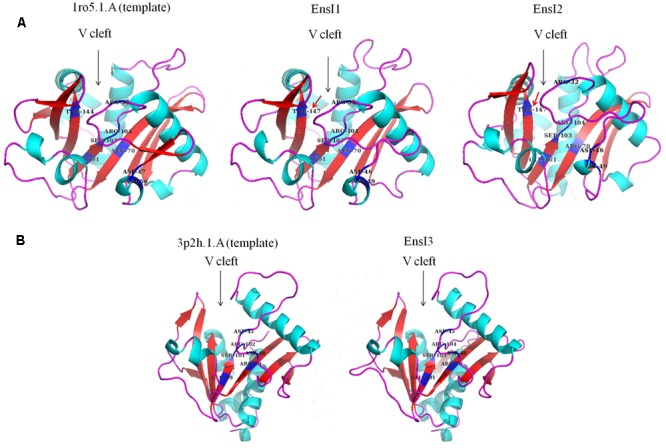
Three dimensional structures of three AHL synthases predicted by SWISS-MODEL. **(A)** The 3D structures of EnsI1 and EnsI2 were based on the experimental crystal structure of LasI of *Pseudomonas aeruginosa* (SMTL id 1ro5.1). Conserved threonine at position 147 was marked with red arrows (THR-147). **(B)** The 3D structure of EnsI3 was based on the crystal structure of TofI of *Burkholderia glumae* (SMTL id 3p2h.1).

Using the protein homology modeling server Swiss-Model and the PyMOL molecular graphics system, we obtained the 3D structures of three AHL synthases. As shown in **Figure 4**, the 3D structures of three AHL synthases of X097 showed similar core elements including the V-shaped cleft active site of the enzyme and multiple conserved residues (ARG-32, ASP-46, ASP-49, ARG-70, GLU-101, SER-103, and ARG-104 in EnsI1 and EnsI2) that are essential for AHL synthesis. However, 3D structures of both EnsI1 and EnsI2 had a conserved threonine at position 147 (THR-147 in **Figure 4A**, marked with red arrows), while there was no threonine at this position in the 3D structure of EnsI3 (**Figure 4B**).

### Lifestyle and Nutrient Affect Growth and AHL Production in X097

Strain X097 was cultured in broth and agar plate to represent planktonic and biofilm lifestyle, respectively (Supplementary Figures S3A,B). X097 grew rapidly both in LB broth and agar plate. After 2 days of cultivation, the colony was large, convex and opaque on LB agar plate (Supplementary Figure S3C), and the OD_600_ reached about 6.5 in broth (Supplementary Figure S4A). However, the growth rate was much slower in NFB medium, especially in NFB broth. The colony was flat and semitransparent on NFB agar plate after 3 days of cultivation (Supplementary Figure S3C). It took 4 days to notice the growth by turbidity in NFB broth. The growth curve of X097 in NFB broth showed maximum density on 8th day, and became stable after the 12th day (Supplementary Figure S4B).

*N*-acyl homoserine lactones production and profiles by X097 in LB or NFB medium with different lifestyles were measured. In LB, X097 produced multiple AHLs both in planktonic and biofilm lifestyle, while 3-oxo-C6-HSL and C6-HSL were additionally detected in biofilm compared with in planktonic lifestyle (**Table 3** and Supplementary Figure S5A). In contrary, when grown in NFB, it produced less AHL molecules (fewer in number). They were identified as C6-HSL and C8-HSL in planktonic lifestyle, and C6-HSL and 3-oxo-C6-HSL in biofilm (**Table 3** and Supplementary Figure S5B).

### Lifestyle Affects the Expression of QS Genes of X097 Grown in LB Medium

**FIGURE 5:**
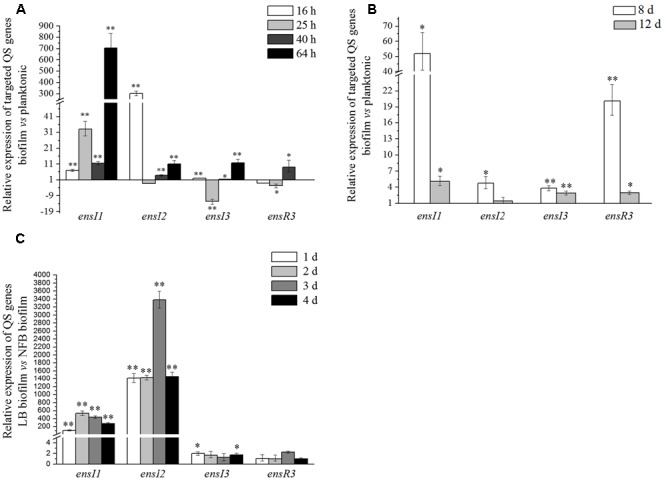
Relative expression levels of *ensI1, ensI2, ensI3*, and *ensR3* in different lifestyles or nutrients. **(A)** Biofilm to planktonic lifestyle in LB medium; **(B)** Biofilm to planktonic lifestyle in NFB medium; **(C)** LB biofilm to NFB biofilm. Error bars represent the standard deviation of the mean and triplicates were performed for each reaction. Mean difference of relative expression level of target gene between different culture conditions was statistically analyzed by ANOVA. “^∗^” and “^∗∗^” represent significant (*p* < 0.05) and extremely significant (*p* < 0.001), respectively.

Real time PCR showed that for *ensI1* and *ensI2* that located on plasmid, their expression levels in biofilms were almost always higher than their planktonic counterparts were (**Figure 5A**). The only exception was at 25 h, when *ensI2* showed nearly the same expression levels between biofilm and planktonic lifestyles (*p* > 0.05). In the time course, the relative expression level of *ensI1* in biofilm to planktonic lifestyle increased from 7 times to 703 times (*p* < 0.001), while the relative expression level of *ensI2* in biofilm to planktonic lifestyle was more than 303 times from the beginning (*p* < 0.001), and declined sharply after that. In general, plasmid-encoded *ensI1* and *ensI2* were significantly up-regulated at mRNA levels in biofilm lifestyle.

For *ensI3* that located on chromosome, its expression level in biofilm was almost the same as its planktonic counterpart at 16 and 40 h. At 25 h, the expression level of *ensI3* in biofilm was 12.5 times lower than in planktonic culture (*p* < 0.001), while 11.6 times higher than in planktonic culture at 64 h (*p* < 0.001) (**Figure 5A**). The expression level of *ensR3* in biofilm was a little down-regulated compared with that of in planktonic culture at 16 and 25 h, but showed an increase later at 40 h (*p* < 0.05). However, at the stationary phase (64 h), both the expression levels of *ensR3* in biofilm and planktonic cultures was rather lower, as indicated by the high *C*_T_ value (> 35). Thus, the relative expression level of *ensR3* at 64 h in bar chart was absent (**Figure 5A**). There was little difference between the mRNA levels in biofilm and planktonic lifestyles for *ensI3* and *ensR3* genes.

### Lifestyle Affects the Expression of QS Genes of X097 Grown in NFB Medium

In NFB medium, less AHL molecules were detected. We were interested in how three AHL synthase genes were expressed. As showed in **Figure 5B**, at 8 days, all QS genes were always up-regulated, but not very much, in biofilm when compared to its planktonic counterparts in NFB medium. It is notable that the ratio of the expression level of *ensI1* in biofilm to planktonic lifestyle was about 50 times at 8 days when the CFU was at the maximum value (*p* < 0.05). On the other hand, at 12 days when the CFU began to stabilize, the ratio of relative expression level of four genes became much close to 1, which suggested that there was slightly difference between the mRNA levels of four genes in biofilm and planktonic lifestyles in stationary phase in NFB medium.

### Nutrient Affects the Expression of QS Genes of X097 Grown in Biofilms

The relative expression level of *ensI1, ensI2, ensI3*, and *ensR3* in LB and NFB biofilm at the same day were compared in order to see the effect of nutrient on the expression of QS genes. Both the expression levels of *ensI1* and *ensI2* were significantly higher in LB biofilm than in NFB biofilm (*p* < 0.001), with great fold changes of 106 to 533 and 1 416 to 3 377, respectively (**Figure 5C**). However, the relative expression level of *ensI3* and *ensR3* in two biofilms were almost the same as each other. In summary, nutrient significantly affected the expression of plasmid-encoded *ensI1* and *ensI2* genes, while had no effect on the expression of chromosome-encoded *ensI3* and *ensR3* genes.

## Discussion

It is known that quite a few bacteria produce multiple AHLs, and possess multiple AHL synthase genes (Sanchez-Contreras et al., 2007; Huang et al., 2009). This sophisticated signaling system helps finely control bacterial activity, enable them to better adapt to their living environments (von Bodman et al., 2008; Papenfort and Bassler, 2016). AHL synthase gene locates either on chromosome or on plasmid. With growing genome database, it is possible to get the statistic information about genetic location of AHL synthase gene. Investigation of the patterns and driving force for the spatial distribution of AHL synthase genes will help understand their phylogenetic development and hence the ecological and evolutionary significance of AHL-based QS system; however, this issue did not catch much attention so far.

Lifestyle and nutrient are two basic and important environmental factors to rhizosphere bacteria. How QS system responses to environmental factors is quite an interesting scientific question (Lee et al., 2013; Baumgardt et al., 2016). In this study, the response of QS system to laboratory lifestyle and nutrients by *E. adhaerens* X097 was monitored in terms of gene expression and chemical profile of QS signal. Our results suggested that plasmid-encoded AHL synthase genes were significantly more sensitive than chromosome-encoded one in respond to different lifestyles or nutrients. The evolution history of plasmid- and chromosome-encoded AHL synthase genes remains unclear. Here we summarized the AHL synthase genes’ locations and presented the phylogenetic clustering of plasmid- and chromosome-encoded *luxI* homologs in *Rhizobiaceae* family. It is noteworthy that there is usually unique AHL synthase gene on chromosome while multiple AHL synthase genes on plasmids. We assume that this may be a selection result of environmental adaptation. Plasmids and insertion sequences are important players in horizontal gene transfer that promotes bacterial adaptation by vectoring ecologically important traits between strains and species (Deng et al., 1995; Harrison and Brockhurst, 2012). The hypersensitive response of plasmid-encoded AHL synthases prompted us to speculate that in order to better adapt to the changing environmental conditions, bacteria might acquire more plasmid-encoded AHL synthase genes along with plasmid transfer between different strains or species in the evolution process. The presence of a potential insertion sequence IS3 in the upstream region of *ensI2* gene (**Figure 2**) supports our explanation.

In terms of AHL chemical profile, QS system was more active in nutrient rich condition than in nutrient deficient condition, which was reflected by multiple AHL signals from LB medium and less from NFB medium. Biofilm lifestyle also affected the AHL profiles both in LB and NFB medium (**Table 3**). In fact, the natural habitats are much more complex and volatile, which inevitably complicated the performance of QS system. One report using *S. meliloti*-alfalfa symbiotic system as a model has demonstrated that the synthesis of short-chain AHL was significantly diminished in nitrogen-fixing bacteroids during nodule development, while the AHL production recommenced by intensively proliferating vegetative forms of bacteria in the senescent and saprophytic zones of nodules (Wielbo et al., 2010). Here we observed differentially expressed AHL signals under different culture conditions. This makes ecological sense since the QS-regulated traits will surely be affected as a consequence. However, linking AHL profile to AHL synthase gene expression is not straightforward, since there is still a long way from mRNA level to AHL production. It was reported that AHL production and accumulation could be affected by many factors at the level of transcription, translation and protein activity (Baumgardt et al., 2014).

In strain X097, plasmid-encoded EnsI1 and EnsI2 belong to G1 and chromosome-encoded EnsI3 belong to G2. EnsI1 and EnsI2 are different from EnsI3 in two ways. Firstly by sequence similarity, and secondly by a conserved threonine at position 147 in protein 3D structure. That also explained why both EnsI1 and EnsI2 produced 3-oxo-C8-HSL while EnsI3 not, since it was reported that for AHL synthases that are known to produce 3-oxo-HSL, the residue at this position is typically a threonine (Watson et al., 2002). Despite large numbers of AHL synthase genes were identified in public genome data in *Rhizobiaceae* family, only the minority has ever been experimentally validated by heterologous expression in *E. coli* (Sanchez-Contreras et al., 2007). The crystal 3D structure determined by X-ray crystallography was only available in fewer AHL synthases (Watson et al., 2002; Gould et al., 2004; Chung et al., 2011). The improvement of these data will provide a basis for better comparing the similarities and differences between plasmid- and chromosome-encoded AHL synthase genes.

Current researches have indicated that *E*. *adhaerens* is an important microorganism in terms of biotechnology, ecology and evolutionary (Rogel et al., 2001; Rudder et al., 2014; Ormeño-Orrillo et al., 2015; Xu et al., 2015). Strain X097 was the first reported AHL-producer in *E*. *adhaerens* (Huang et al., 2013a). In this study, we report the draft genome of strain X097 and validate the functions of three predicted *luxI* homologs through inducible heterologous expression in *E. coli*. This is the first comprehensive study of multiple QS elements in *E. adhaerens* and their response to changing culture conditions. We demonstrate that both lifestyle and nutrient significantly affect the expression of AHL synthase genes and AHL production in *E*. *adhaerens* X097. We provide evidence that there is an inconsistence response of plasmid- and chromosome-encoded AHL synthase genes to different lifestyles or nutrients. This is practically important when *E. adhaerens* is applied in plant transformation technology (Rudder et al., 2014). Our findings help to understand how bacteria utilize multiple QS system to cope with changing environments. In addition, the comprehensive phylogenetic analysis place AHL synthase genes to different groups, which may help to elucidate the evolutionary history of this gene in *Rhizobiaceae.*

## Author Contributions

YZ conducted most of the experiments, analyzed the data, and wrote the manuscript. YW contributed to the experiment in NFB medium. ZY contributed to the experimental design, technical guidance of quantitative real time PCR and manuscript revising. YH conceived and design this study, and was in charge of the technical support, data analysis and manuscript writing. All authors agree to be accountable for the content of the work.

## Conflict of Interest Statement

The authors declare that the research was conducted in the absence of any commercial or financial relationships that could be construed as a potential conflict of interest.

## Footnotes

1 http://soap.genomics.org.cn/soapdenovo.html

2 http://swissmodel.expasy.org/
