# Supplementary material for: Hypersensitive Response of Plasmid-Encoded AHL Synthase Gene to Lifestyle and Nutrient by Ensifer adhaerens X097
Source: Front Microbiol. 2017 Jun 28;8:1160. doi: 10.3389/fmicb.2017.01160 (PMC5487405; doi:10.3389/fmicb.2017.01160)
Supplement: Supplementary file 1 [file Table_1.PDF]

Supplementary Table S1 Primers used in this study.

primers	Sequence 5’-3’	Target gene/description
AI1F	5’-CGCGAATTCTGTGCAGATTCTCGCAATTTC-3’	cloning of *ensI1*
AI1R	5’-CGCAAGCTTAAAGGGGTGGATGATATTGG-3’	
AI2F	5’-CGCGGATCCGATGGAGATATTAGCTGTGCC-3’	cloning of *ensI2*
AI2R	5’-CGCAAGCTTAGCAACGGAGTTGTGGTGCA-3’	
AI3F	5’-CGCGAATTCTATGATCAGGATCCTGAACGG-3’	cloning of *ensI3*
AI3R	5’-CGCAAGCTTGGCGGCGCGGGCGGTTTCCA-3’	
16SF	5’-GAATAACGCAGGGAAACTT-3’	housekeeping gene
16SR	5’-TGATCATCCTCTCAGACCA-3’	
AI1 40F	5’-GGAGATCAGGCTTCTCTACA-3’	real time RCR of *ensI1*
AI1 135R	5’-GTGCGTCGAAACAATCCGAT-3’	
AI2 37F	5’-GGAGAATTTGCTGACTTG-3’	real time RCR of *ensI2*
AI2 135R	5’-TTGATCGTAAGCATCGACC-3’	
AI3 136F	5’-TCGACAACTACGACCAGGCA-3’	real time RCR of *ensI3*
AI3 272R	5’-TTTCCGGATTATCGCCAAGC-3’	
LuxR 406F	5’-TGCTCGCAATCAACGTCAAC-3’	real time RCR of *ensR3*
LuxR 538R	5’-ATCTCTCGGGTCGAAATGCT-3’	
