# Supplementary material for: Hypersensitive Response of Plasmid-Encoded AHL Synthase Gene to Lifestyle and Nutrient by Ensifer adhaerens X097
Source: Front Microbiol. 2017 Jun 28;8:1160. doi: 10.3389/fmicb.2017.01160 (PMC5487405; doi:10.3389/fmicb.2017.01160)
Supplement: Supplementary file 2 [file Table_2.PDF]

**Supplementary Table S2** Sequence similarity matrix of LuxI homologs. The accession number of each AHL synthase was indicated in brackets.

| | X097 EnsI1 | X097 EnsI2 | X097 EnsI3 | CA EnsI2 | CA LasI | OV14 EnsI1 | OV14 LasI |
|---|---|---|---|---|---|---|---|
| X097 EnsI1 (AFN06796) | ID | 56.4% | 25.7% | 55.2% | 25.7% | 49.5% | 23.0% |
| X097 EnsI2 (AFN06798) | | ID | 27.4% | 90.9% | 27.4% | 57.0% | 35.7% |
| X097 EnsI3 (KP966548) | | | ID | 26.6% | 100% | 27.4% | 89.3% |
| CA EnsI2 (KDP72381) | | | | ID | 26.6% | 57.1% | 35.7% |
| CA LasI (KDP74535) | | | | | ID | 27.4% | 89.3% |
| OV14 EnsI1 (AHK46650) | | | | | | ID | 27.6% |
| OV14 LasI (AHK44523) | | | | | | | ID |
