# Supplementary material for: Hypersensitive Response of Plasmid-Encoded AHL Synthase Gene to Lifestyle and Nutrient by Ensifer adhaerens X097
Source: Front Microbiol. 2017 Jun 28;8:1160. doi: 10.3389/fmicb.2017.01160 (PMC5487405; doi:10.3389/fmicb.2017.01160)

Supplementary Figure S1 Biosensor assay of AHL signals heterologous expressed by three AHL synthases EnsI1, EnsI2 and EnsI3 (A), and TLC analysis of AHL signals heterologous expressed by EnsI1 and EnsI2 (B). C^+, C8-HSL positive control; C^-, EA negative control. M1-M3, AHL markers.

A

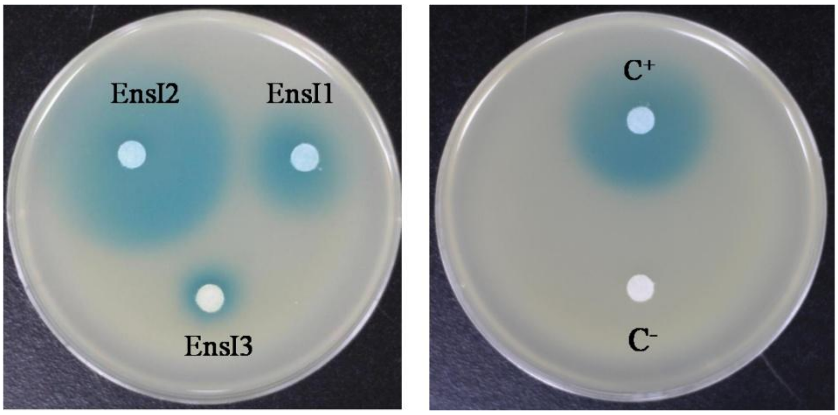

B

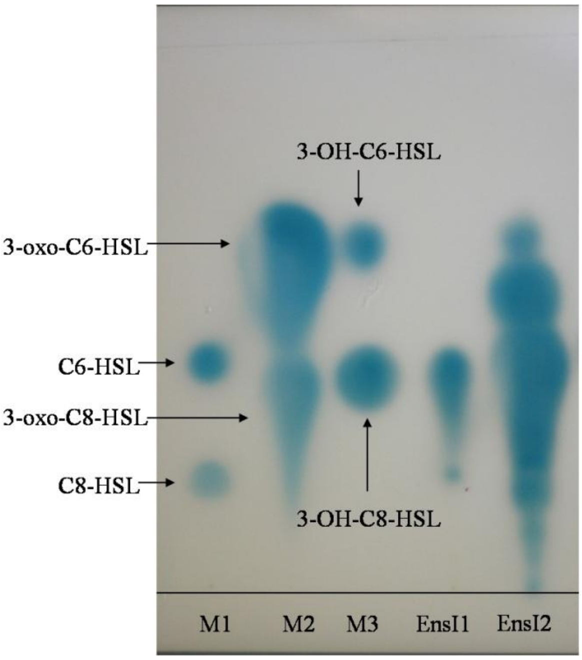

Supplement: Supplementary file 3 [file Image_1.PDF]
