# Supplementary material for: Hypersensitive Response of Plasmid-Encoded AHL Synthase Gene to Lifestyle and Nutrient by Ensifer adhaerens X097
Source: Front Microbiol. 2017 Jun 28;8:1160. doi: 10.3389/fmicb.2017.01160 (PMC5487405; doi:10.3389/fmicb.2017.01160)

Supplementary Figure S2 Representative GC-MS analysis of AHL signals produced by EnsI1 (A), EnsI2 (B) and EnsI3 (C). LB medium was used to grow bacteria for the AHL extractions.

A

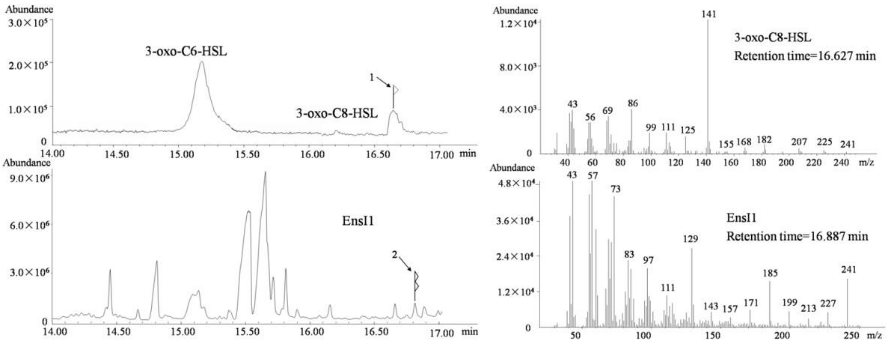

B

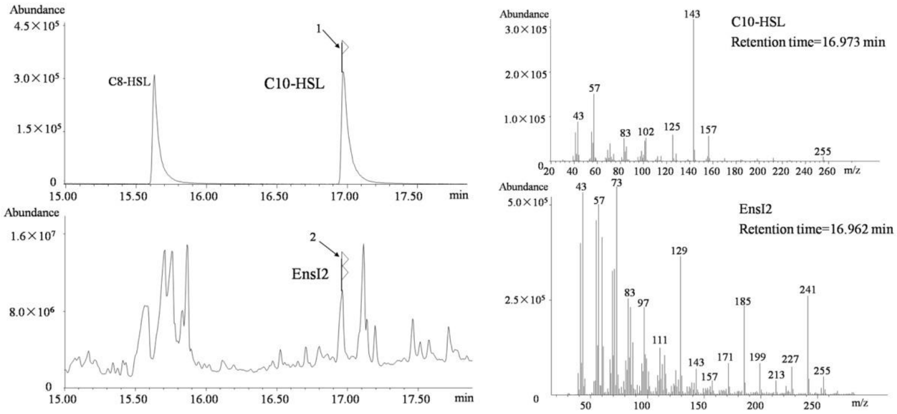

C

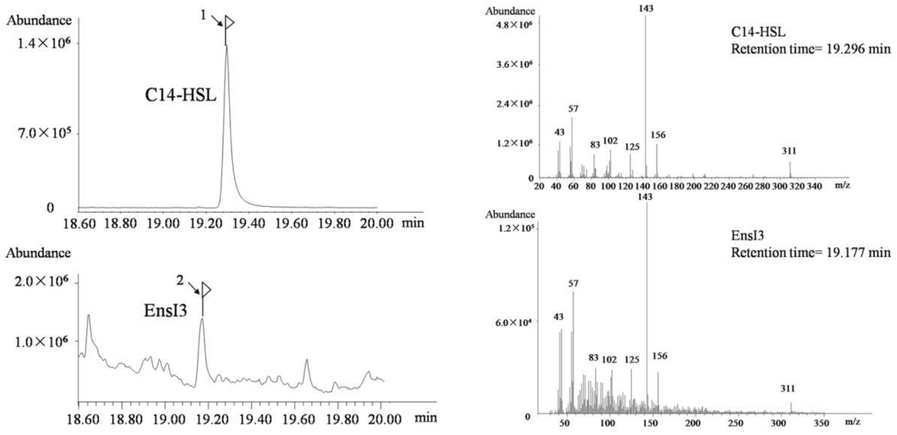

Supplement: Supplementary file 4 [file Image_2.PDF]
