# Supplementary material for: Hypersensitive Response of Plasmid-Encoded AHL Synthase Gene to Lifestyle and Nutrient by Ensifer adhaerens X097
Source: Front Microbiol. 2017 Jun 28;8:1160. doi: 10.3389/fmicb.2017.01160 (PMC5487405; doi:10.3389/fmicb.2017.01160)
Supplement: Supplementary file 5 [file Image_3.PDF]

Supplementary Figure S3 The growth of X097 in different media with different lifestyles. Planktonic and biofilm lifestyle of X097 in LB medium (A) and NFB medium (B). Colony morphology of X097 streaked on LB (2 d) or NFB (3 d) agar plates (C).

A

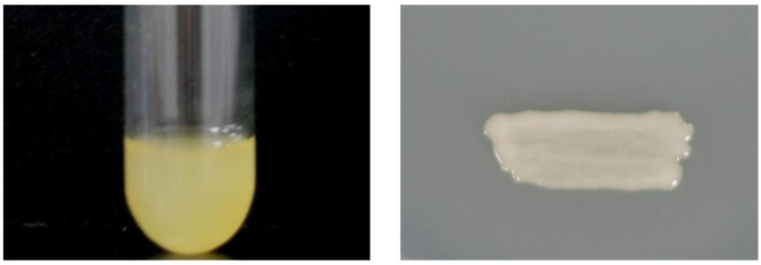

planktonic biofilm

B

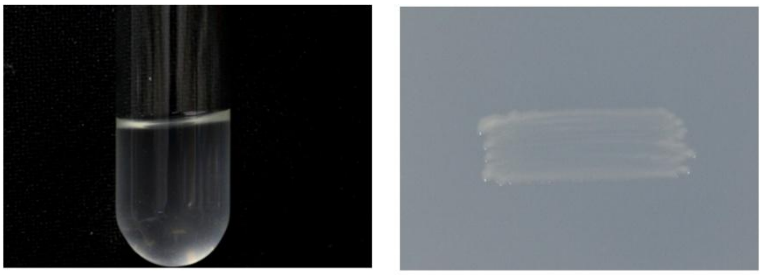

planktonic biofilm

C

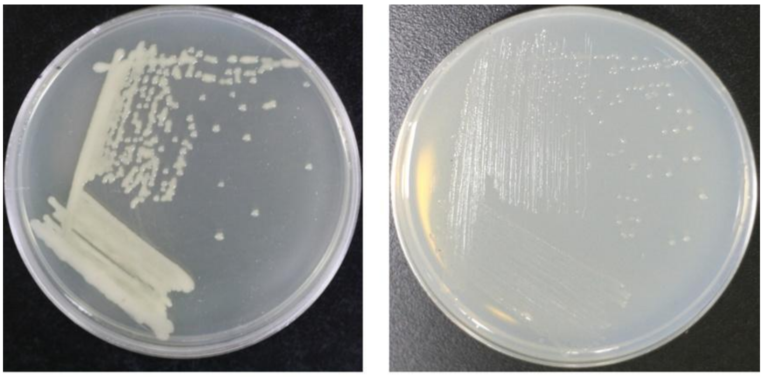

LB NFB
