# Supplementary material for: Hypersensitive Response of Plasmid-Encoded AHL Synthase Gene to Lifestyle and Nutrient by Ensifer adhaerens X097
Source: Front Microbiol. 2017 Jun 28;8:1160. doi: 10.3389/fmicb.2017.01160 (PMC5487405; doi:10.3389/fmicb.2017.01160)

Supplementary Figure S4 Growth curve of X097 in LB broth (A) and NFB broth (B) $\log_{10}$ (CFU)/mL. Error bars represent the standard deviation of the mean.

A

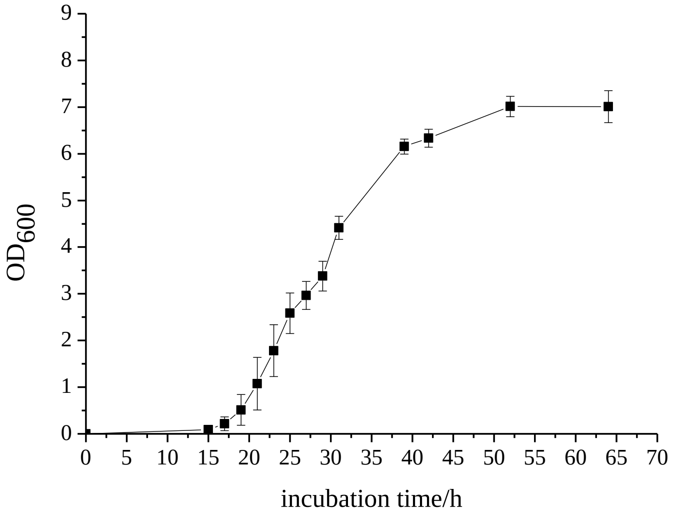

B

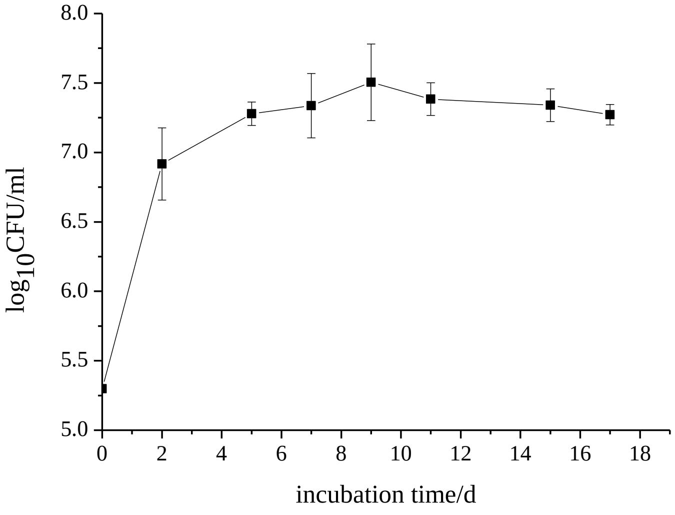

Supplement: Supplementary file 6 [file Image_4.PDF]
