# Supplementary material for: Hypersensitive Response of Plasmid-Encoded AHL Synthase Gene to Lifestyle and Nutrient by Ensifer adhaerens X097
Source: Front Microbiol. 2017 Jun 28;8:1160. doi: 10.3389/fmicb.2017.01160 (PMC5487405; doi:10.3389/fmicb.2017.01160)

Supplementary Figure S5 TLC analysis of AHL profiles of X097 cultured in planktonic or biofilm lifestyle in LB (A) and NFB medium (B). M1-M3: AHL standard markers. All AHLs were extracted from stationary phase cultures.

A

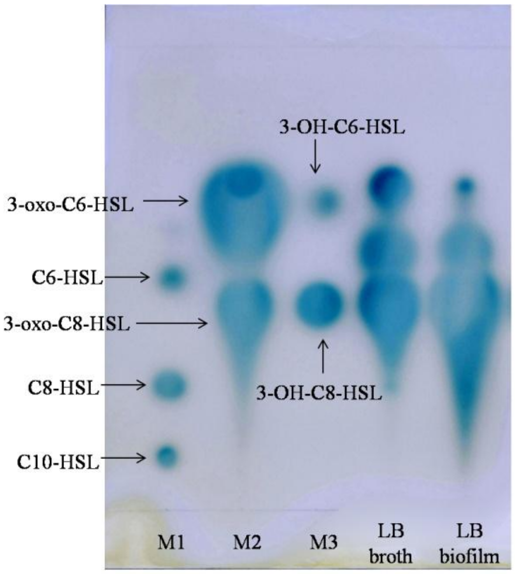

B

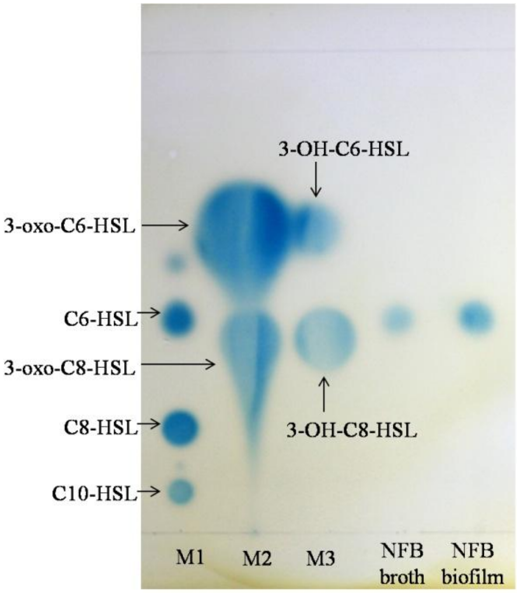

Supplement: Supplementary file 7 [file Image_5.PDF]
